# Integrative Analysis of Siglec-15 mRNA in Human Cancers Based on Data Mining

**DOI:** 10.7150/jca.38747

**Published:** 2020-02-10

**Authors:** Qiu-ting Li, Zao-zao Huang, Yao-bin Chen, Hong-yi Yao, Zun-hui Ke, Xiao- xiao He, Meng-jun Qiu, Meng-meng Wang, Zhi-fan Xiong, Sheng-li Yang

**Affiliations:** 1 Division of Gastroenterology, Liyuan Hospital, Tongji Medical College, Huazhong University of Science and Technology, Wuhan 430077, China.; 2 Yangchunhu community Hospital, Liyuan Hospital, Tongji Medical College, Huazhong University of Science and Technology, Wuhan 430077, China.; 3 Institute of Pathology, Tongji Hospital, Tongji Medical College, Huazhong University of Science and Technology, Wuhan 430030, China.; 4 Department of Rehabilitation, Liyuan Hospital, Tongji Medical College, Huazhong University of Science and Technology, Wuhan 430077, China.; 5 Wuhan Children's Hospital (Wuhan Maternal and Child Healthcare Hospital), Tongji Medical College, Huazhong University of Science & Technology, Wuhan 430015, China.; 6 Cancer Center, Union Hospital, Tongji Medical College, Huazhong University of Science and Technology, 1277 JieFang Avenue, Wuhan 430022, China.

**Keywords:** Siglec-15, cancer, immunotherapy, biomarker

## Abstract

**Objective**: Cancer is expected to be the leading cause of death worldwide within the 21st century and is the single most important obstacle to extending life expectancy. Unfortunately, the most effective approach to combating cancers remains a complex and unsolved problem. Siglec-15 is a member of the Siglec family and plays a conserved regulatory role in the immune system of vertebrates. Previous studies on Siglec-15 have focused on its function in osteoclast regulation. The purpose of this study was to explore the significance of Siglec-15 mRNA in human cancer mainly based on information obtained from online databases.

**Method**: Data were collected from several online databases. Serial analysis of gene expression (SAGE) and Virtual Northern, UALCAN Database Analysis, Catalog of Somatic Mutations in Cancer (COSMIC) analysis, the cBio cancer genomics portal, Cancer Regulome tools and data, Kaplan-Meier Plotter Analysis and the UCSC Xena website were used to analyze the data.

**Results**: Compared with normal tissues, Siglec-15 up-regulation was widely observed in tuomrs. Differences in Siglec-15 expression were associated with different prognoses. Siglec-15 mutations are widely observed in tumors and interact with different genes in different cancer types.

**Conclusion**: Siglec-15 is a potential target for the expansion of cancer immunotherapy.

## Introduction

With the development of medical science, novel methods for the diagnosis and treatment of many diseases are being discovered and bring greater opportunities and increased hope to patients worldwide. However, the most effective approach to combating cancer remains a complex and unsolved problem for not only doctors but also scientists. According to the Global Cancer Statistics 2018 report, there were an estimated 18.1 million new cancer cases (17.0 million excluding nonmelanoma skin cancer) and 9.6 million cancer deaths (9.5 million excluding nonmelanoma skin cancer) in 2018 1. The report also stated that cancer is expected to be the leading cause of death worldwide within the 21st century and is the single most important obstacle to extending life expectancy 1. Traditional treatments for cancer include surgery, chemotherapy and radiotherapy. Immunotherapy and targeted therapies have also been applied gradually in the clinic, but limitations related to cancer diagnosis and drug resistance remain. To solve these problems, potential biomarkers are needed for the development of novel diagnostic and treatment approaches.

The sialic acid-binding immunoglobulin-like lectin (Siglec) family is the known largest group of vertebrate lectins that can recognize sialylated glycans. Originally, Siglec family members were found on immune cells and determined to be involved in coupling glycan recognition to immunological regulation 2. Siglec-15 is a member of the Siglec family and is considered to play a conserved, regulatory role in the immune system of vertebrates 2. The extracellular domain of Siglec-15 consists of an immunoglobulin variable region and a type 2 constant region (IgC2), which has more than 30% homology with the B7 gene family. These results indicate that Siglec-15 may have similar immunomodulatory functions with the B7 family 3, which includes the renowned immunological therapy biomarker PD-L1. Siglec-15 has been reported to activate the AKT pathway through DAP12 4. The activation of AKT could promote tumor cell proliferation 5-7. Previous studies on Siglec-15 have focused on its function in osteoclast regulation 4, 8, 9. However, some recent studies have demonstrated that Siglec-15 is highly expressed in some cancers and might play important roles in tumor immunity 10, 11.

Few studies have focused on the role of Siglec-15 in tumors, and thus, its expression in tumors remains unclear. To elucidate the role of Siglec-15 in tumors, in this study, database tools were used to obtain information from well-known online databases, such as TCGA, GEO and EGA, to examine Siglec-15 mRNA expression in different types of solid tumors.

## Material & Methods

### Serial Analysis of Gene Expression (SAGE) and Virtual Northern

SAGE is a useful technique developed for the genome-wide analysis of gene expression. It utilizes efficient computational tools to acquire and analyze tag sequences from raw sequence files to compare tag abundances between different libraries 12. Monochromatic SAGE/cDNA Virtual Northern displays the expression of genes in all human organs. In this study, both SAGE and Monochromatic SAGE/cDNA Virtual Northern tools from the Cancer Genome Anatomy Project (https://cgap.nci.nih.gov/Genes) were used. The SAGE results revealed the expression of Siglec-15 in cancer and normal tissues, and the Virtual Northern results displayed the levels of Siglec-15 in human tissues.

### UALCAN Database

UALCAN (http://ualcan.path.uab.edu) is a comprehensive, user-friendly, and interactive web resource for analyzing cancer OMICS data. The database uses 31 types of cancer RNA-seq and clinical information from TCGA to complete in-depth analyses of TCGA gene expression data, including analysis of the relative expression of a query gene across tumor and normal samples 13. In this study, the UALCAN database was used to obtain data from the TCGA database and compare the expression of Siglec-15 mRNA in tumors and normal tissues.

### Catalog of Somatic Mutations in Cancer (COSMIC)

COSMIC (https://cancer.sanger.ac.uk/cosmic/) is the world's largest and most comprehensive resource for exploring the impact of somatic mutations in human cancer. It has a collection of data on millions of coding mutations, noncoding mutations, genomic rearrangements, fusion genes, copy number abnormalities and gene expression variants in the human genome all in one database so that researchers can explore these data more easily^14^. In this study, COSMIC was used to investigate the mutations of Siglec-15 in human cancers, and the results are depicted in pie charts.

### Mutation Rate and Distribution of Different Exons

The cBio cancer genomics portal (http://cbioportal.org) is an open-access resource that simplifies the molecular analysis of cancer tissues and cell lines into easy-to-understand genetic, epigenetic, gene expression, and proteomic events^15^, ^16^. We used cBioPortal to analyze Siglec-15 in TCGA pan-cancer data to identify coexpressed genes.

### Integrative Data Visualization

The Cancer Regulome tools and data (http://explorer.cancerregulome.org/) from the TCGA database were used to create circus plots to display the expression of Siglec-15 and its correlation with other genes in tumors. Spearman correlation was used to show the pairwise correlation between two genes. Only genes with P values > - log10 are shown in the circus plots.

### Kaplan-Meier Plotter

The Kaplan-Meier Plotter (http://kmplot.com/analysis/) is an online tool that is typically used to draw survival curves. The system includes gene chip and RNA-seq data, and sources for the databases include GEO, EGA, and TCGA 17. In this study, the mRNA expression levels of Siglec-15 in each cancer were stratified into high and low groups by the web, and then survival curves were drawn. Hazard ratios with 95% confidence intervals and log-rank P values were also calculated.

### Genomic Data and Views

The UCSC Xena website (http://xena.ucsc.edu/) offers tools for the visualization and exploration of TCGA genomic data 18. According to the coexpression results from Oncomine and an interesting study 11, we performed a search on Siglec-15, SMAD7, ATP5A1 and CD274 (PD-L1) using the Genes viewing mode.

## Results

### Siglec-15 mRNA in Normal and Tumor Tissues

**Figure 1:**
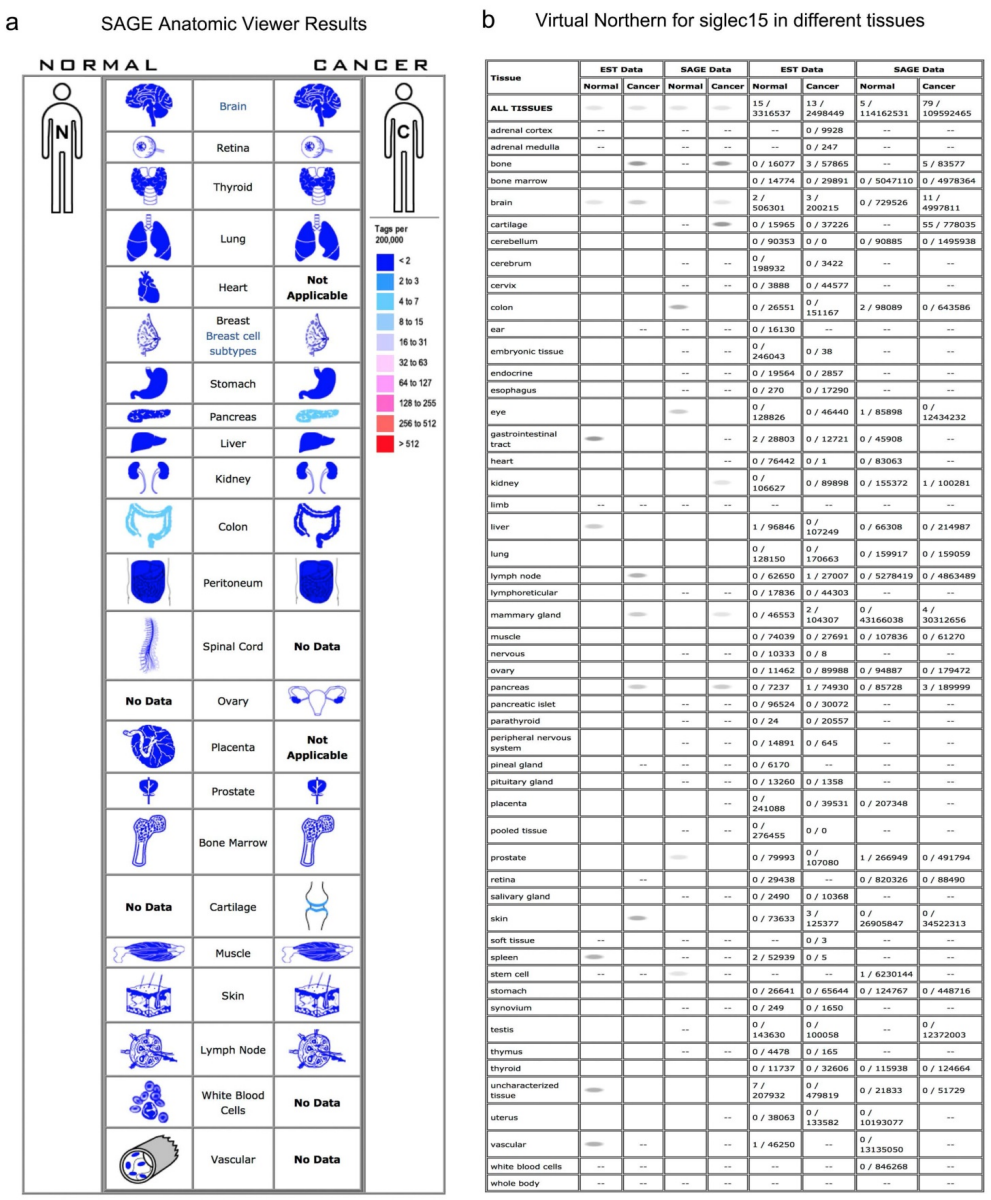
The SAGE Anatomic Viewer. a. Expression profile for Siglec-15 in human cancers displayed by the SAGE Digital Gene Expression Displayer (DGED). b Monochromatic SAGE/cDNA Virtual Northern results for Siglec-15 in different tissues.

Normally, Siglec-15 has low expression in nonimmune organs. As shown in the SAGE results in Figure 1a, a higher expression of Siglec-15 was observed in pancreatic cancer, cartilage tumors and normal colon tissues. In the Virtual Northern results, compared with normal tissues, Siglec-15 was upregulated in bone, breast and pancreatic cancer tissues (Figure 1b).

### Upregulation of Siglec-15 mRNA in Cancer

**Figure 2:**
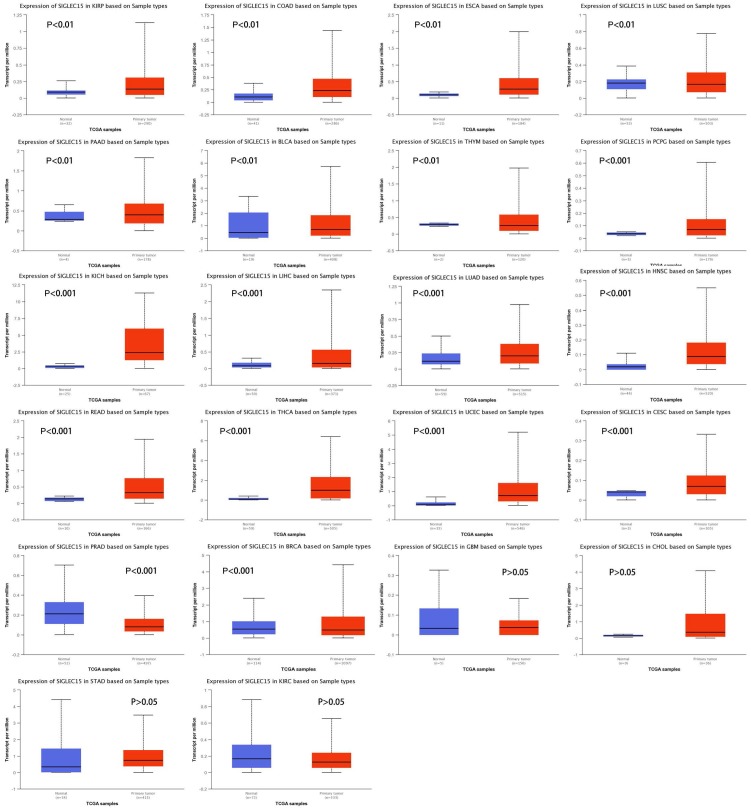
Siglec-15 mRNA was evaluated in human cancers by UALCAN database analysis.

To examine Siglec-15 mRNA levels across different cancer types, data from the UALCAN database were used. We chose TCGA data as the data source and compared the Siglec-15 mRNA levels in cancer tissues with those in normal tissues. As shown in Figure 2, the expression of Siglec-15 mRNA in most cancers was obviously different from that in normal tissues. Higher Siglec-15 mRNA levels were found in kidney renal papillary cell carcinoma (KIRP), colon adenocarcinoma (COAD), esophageal carcinoma (ESCA), pancreatic adenocarcinoma (PAAD), bladder urothelial carcinoma (BLCA), pheochromocytoma and paraganglioma (PCPG), kidney chromophobe (KICH), liver hepatocellular carcinoma (LIHC), lung adenocarcinoma (LUAD), head and neck squamous cell carcinoma (HNSC), rectum adenocarcinoma (READ), thyroid carcinoma (THCA), uterine corpus endometrial carcinoma (UCEC) and cervical squamous cell carcinoma (CESC),while downregulation could be observed in thymoma (THYM), lung squamous cell carcinoma (LUSC), prostate adenocarcinoma (PRAD) and breast invasive carcinoma (BRCA). However, no significant differences were observed in glioblastoma multiforme (GBM), cholangiocarcinoma (CHOL), stomach adenocarcinoma (STAD) and kidney renal clear cell carcinoma (KIRC).

### Siglec-15 Mutations in Cancer

**Figure 3:**
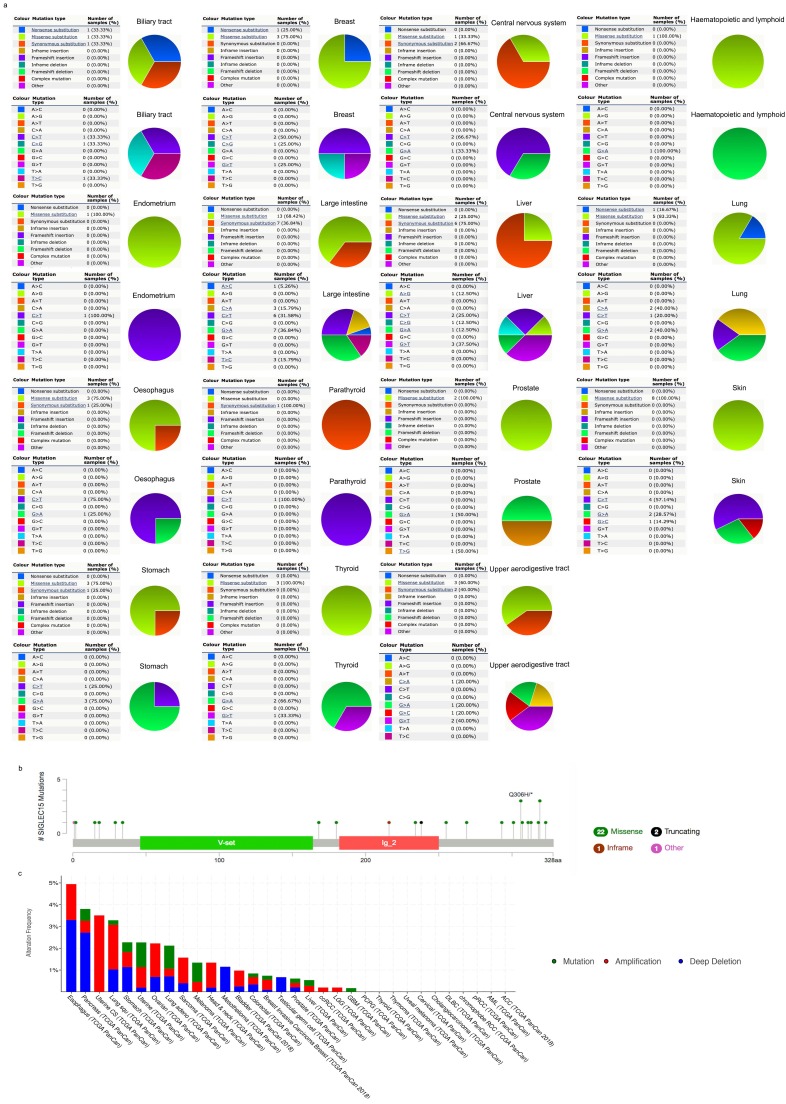
(a) Pie chart showing the percentage of the different mutation types of Siglec-15 in human cancers according to the COSMIC database. (b) Mutation diagram of Siglec-15 in different cancer types across protein domains. (c) Siglec-15 mutation level in the TCGA cancer database.

COSMIC provided information on Siglec-15 mutations in different cancers, which included substitution missense, nonsense and synonymous mutations, and the results are depicted in pie charts. Nonsense substitutions were found in biliary tract cancer (33.33%), breast cancer (25%) and lung cancer (16.67%), while substitution missense mutations were observed in biliary tract cancer (33.33%), breast cancer (75%), central nervous system cancer (33.33%), hematopoietic and lymphoid cancer (100%), endometrial cancer (100%), large intestine cancer (68.42%), liver cancer (25%), lung cancer (83.33%), esophageal cancer (75%), prostate cancer (100%), skin cancer (100%), stomach cancer (75%), thyroid cancer (100%) and upper aerodigestive tract cancer (60%). Additionally, synonymous substitution mutations appeared in biliary tract cancer (33.33%), central nervous system cancer (66.67%), large intestine cancer (36.84%), liver cancer (75%), esophageal cancer (25%), parathyroid cancer (100%), stomach cancer (25%) and upper aerodigestive tract cancer (40%). C>T and G>A mutations were most common in the Siglec-15 coding strand, both of which were observed in eleven cancer types. A>T and T>A mutations in Siglec-15 were not found in the TCGA cancer samples. Other types of base mutations occurred sporadically in different cancers (Figure 3a). With the help of cBioPortal, we found that a total of 22 mutation sites were detected and located between amino acids 0 and 328 (Figure 3b). Figure 3c shows the mutation results from cBioPortal, which shows the Siglec-15 mutation level in the TCGA cancer database. Pancreatic cancer, lung cancer, stomach cancer, uterine cancer, melanoma cancer, colorectal cancer, breast cancer, prostate cancer, liver cancer and glioblastoma multiforme all had high mutation levels.

### Genome-wide Association of Siglec-15 mRNA in Cancer

**Figure 4:**
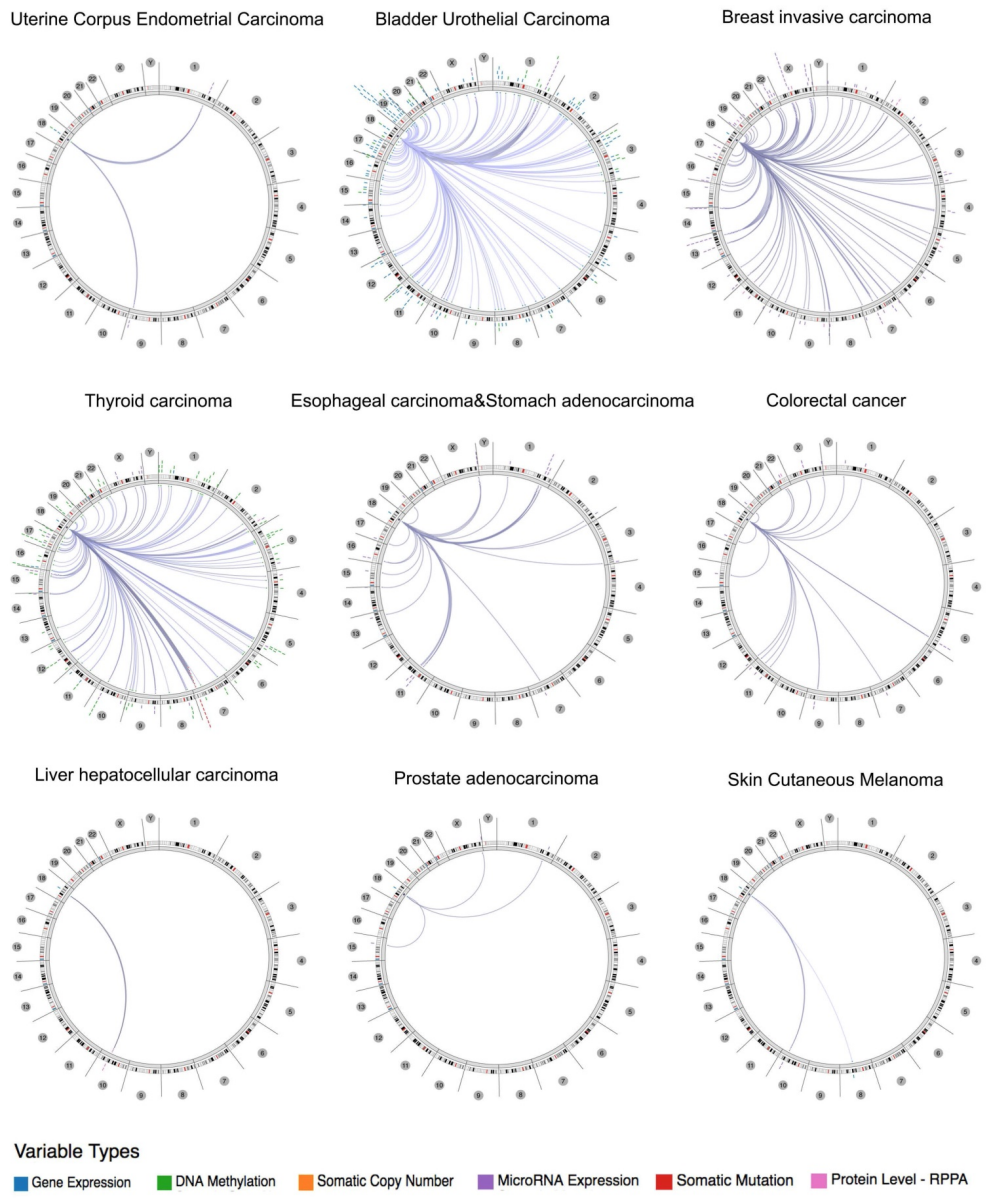
The correlation between Siglec-15 and other genes from the TCGA database (Regulome program).

Using the Regulome Explorer, we further analyzed the relevant human genome location and the correlation between certain genes and Siglec-15 in human cancer. Based on the association among genes, DNA methylation, somatic copy number, somatic mutation and protein level, circus plots were drawn to display the interrelation between Siglec-15 and other genes. According to the data from TCGA, Siglec-15 was associated with other genes that could be detected in colorectal cancer, bladder urothelial carcinoma, breast invasive carcinoma, esophageal carcinoma and stomach adenocarcinoma, uterine corpus endometrial carcinoma, thyroid carcinoma, liver hepatocellular carcinoma, prostate adenocarcinoma and skin cutaneous melanoma (Figure 4). Detailed data can be found in Supplementary Tables 1 to 9.

### Siglec-15 and the Survival Rate of Cancers

**Figure 5:**
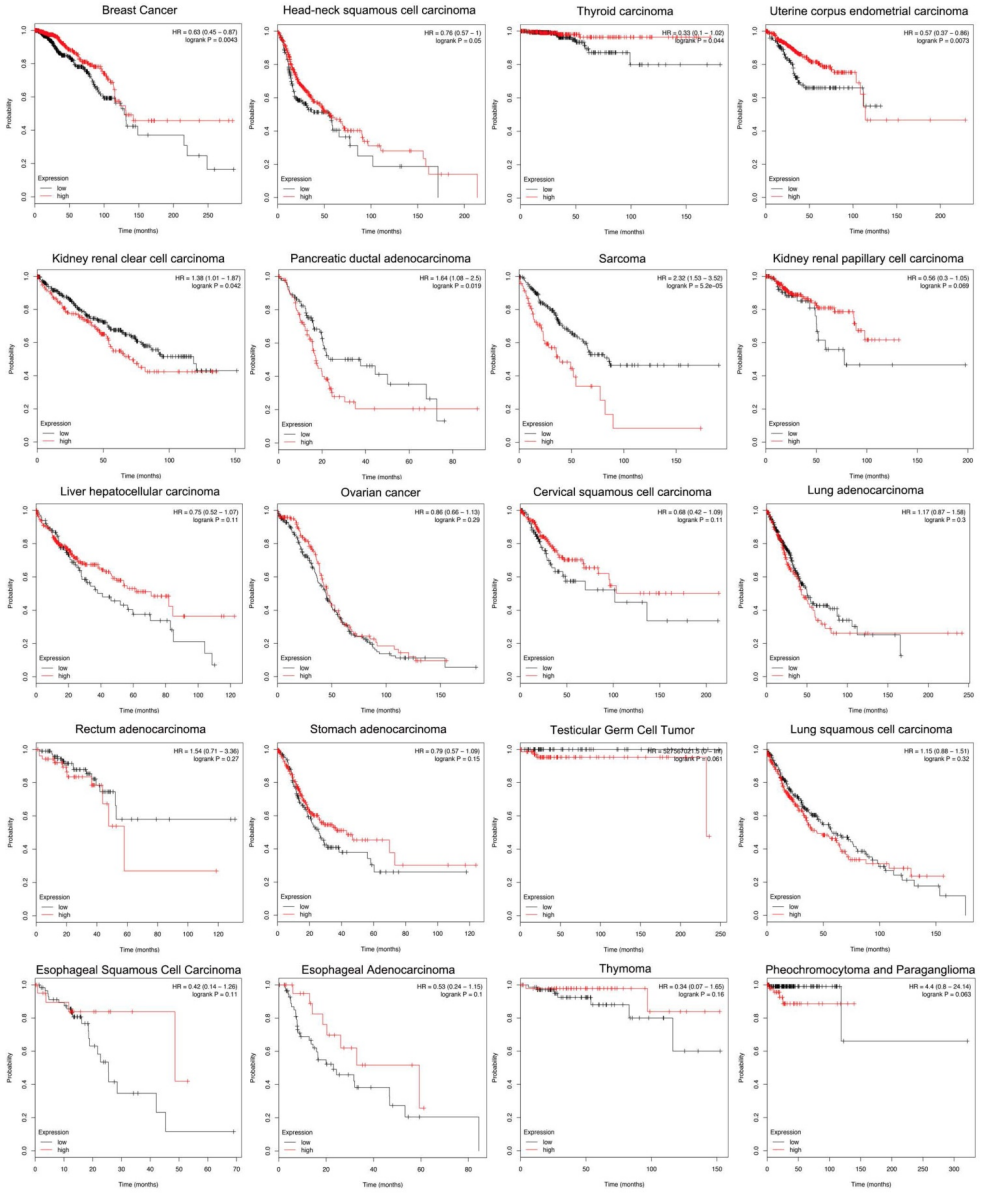
Different expression levels of Siglec-15 mRNA will result in different overall survival rates in cancers (Kaplan-Meier analysis).

According to the Kaplan-Meier analysis results, higher levels of Siglec-15 mRNA indicated worse overall survival in pancreatic ductal adenocarcinoma (P=0.019), sarcoma (P=5.2E-05) and kidney renal clear cell carcinoma (P=0.042). However, the opposite result was observed in breast cancer (P=0.0043), head and neck squamous cell carcinoma (P=0.05), thyroid carcinoma (P=0.044), and uterine corpus endometrial carcinoma (P=0.0073). The expression of Siglec-15 mRNA level have no significant influence in kidney renal papillary cell carcinoma (P=0.069), liver hepatocellular carcinoma (P=0.11), ovarian cancer (P=0.29), cervical squamous cell carcinoma (P=0.11), lung adenocarcinoma (P=0.3), rectum adenocarcinoma (P=0.27), stomach adenocarcinoma (P=0.15), testicular germ cell tumor (P=0.061), lung squamous cell carcinoma (P=0.32), esophageal squamous cell carcinoma (P=0.11), pheochromocytoma and paraganglioma (P=0.063), esophageal adenocarcinoma (P=0.1) and thymoma (P=0.16) (Figure 5).

### Correlations among Siglec-15, SMAD7, ATP5A1, and CD274 (PD-L1)

Based on the results of the Oncomine coexpression analysis and a newly published study 11, we chose SMAD family member 7 (SMAD7), ATP synthase, H+ transporting, mitochondrial F1 complex, α subunit 1, cardiac muscle (ATP5A1), and CD274 (PD-L1) in this study. According to the image created by UCSC Xena, in the TCGA tumor samples, the DNA copy numbers of Siglec-15, SMAD7 and ATP5A1 showed a similar trend, while Siglec-15 and CD274 (PD-L1) showed the opposite trend.

## Discussion

The Siglec family is a group of lectins with a structure containing an N-terminal V-set Ig-like domain and a sialic acid-binding domain, followed by variable numbers of C2-set Ig-like domains. Eleven family members were found to bind sialic acid on glycoconjugates 19. Although their structures are similar, their binding specificity differs, and they bind specific sialoglycoconjugates 20. Most Siglec family members are observed on only hematopoietic cells, as they have very low expression in other tissues, but a recent study found that their expression level changed in some tumor cells 21-23. Several lines of evidence have shown that interactions with sialic acid-binding receptors can influence cancer progression; for example, hypersialylation can induce changes in the physical properties of tumor cells and potentiate the evasion of apoptosis in cancer cells 24, 25. As the Siglec family has the ability to bind specific sialoglycoconjugates, it was regarded as a potential targeted therapy biomarker 26.

Siglec-15 is a Siglec gene family member with the ability to bind the sialyl-Tn antigen, and it has been used as a targeted therapy biomarker for osteoporosis 4, 9, 27, 28. Siglec-15 is an I-type transmembrane protein that includes two immunoglobulin-like domains, a transmembrane domain containing lysine residues and a short cytoplasmic tail. It associates with the activating adaptor proteins DNAX activation protein (DAP)12 and DAP10 via its lysine residue in the transmembrane domain, implying that it functions as an activating signaling molecule. Moreover it is the second human Siglec identified to have an activating signaling potential 2. As Figure 1 shows, Siglec-15 mRNA has very low expression in normal human tissues, including most immune cell types; however, it could be detected in macrophages, bone tumors and pancreatic cancer. A study published in *Nature* proved that Siglec-15 expression was increased in many human cancers; we also obtained the same results, as shown in Figure 2. Compared with normal tissues, the expression of Siglec-15 was upregulated at different levels in KIRP, COAD, ESCA, PAAD, BLCA, PCPG, KICH, LIHC, LUAD, HNSC, READ, THCA, UCEC and CESC. Moreover, a previous study confirmed that Siglec-15 could suppress antigen-specific T cell responses in vitro and in vivo. Additionally, knockdown of Siglec-15 expression did not cause obvious physical abnormalities but did inhibit tumor growth 11. Based on these results, Siglec-15 is believed to be a good potential targeted therapy biomarker.

Since no previous studies have focused on Siglec-15 mutations in human cancers, we explored this topic with the help of COSMIC and cBioPortal. As shown in Figure 3, the results from TCGA demonstrated that Siglec-15 mutations occurred widely in human cancers. The most common type of Siglec-15 mutation was missense substitution, which could be observed in all tumors with mutations. At the base-pair level, C>T and G>A mutations were the most widely observed in tumors. Base substitution can lead to abnormal transcription and translation, which might cause changes in both function and phenotype. Figure 5 shows that a higher level of Siglec-15 expression in pancreatic ductal adenocarcinoma, sarcoma and kidney renal clear cell carcinoma indicates worse overall survival, while the opposite result is true in breast cancer, head and neck squamous cell carcinoma, thyroid carcinoma, and uterine corpus endometrial carcinoma. The reason for this phenomenon is still unclear, but the results shown in Figure 5 suggest that patients with different Siglec-15 expression levels have distinct overall survival rates. Nevertheless, Siglec-15 was found to be a useful biomarker for predicting prognosis. Considering the results presented in Figure 4, we postulate that the reason for the opposing results could be that Siglec-15 interacts with entirely different genes in different types of cancers. Alterations in the expression of Siglec-15 may cause a variety of gene changes across different cancers, which could lead to remarkably different results.

**Figure 6:**
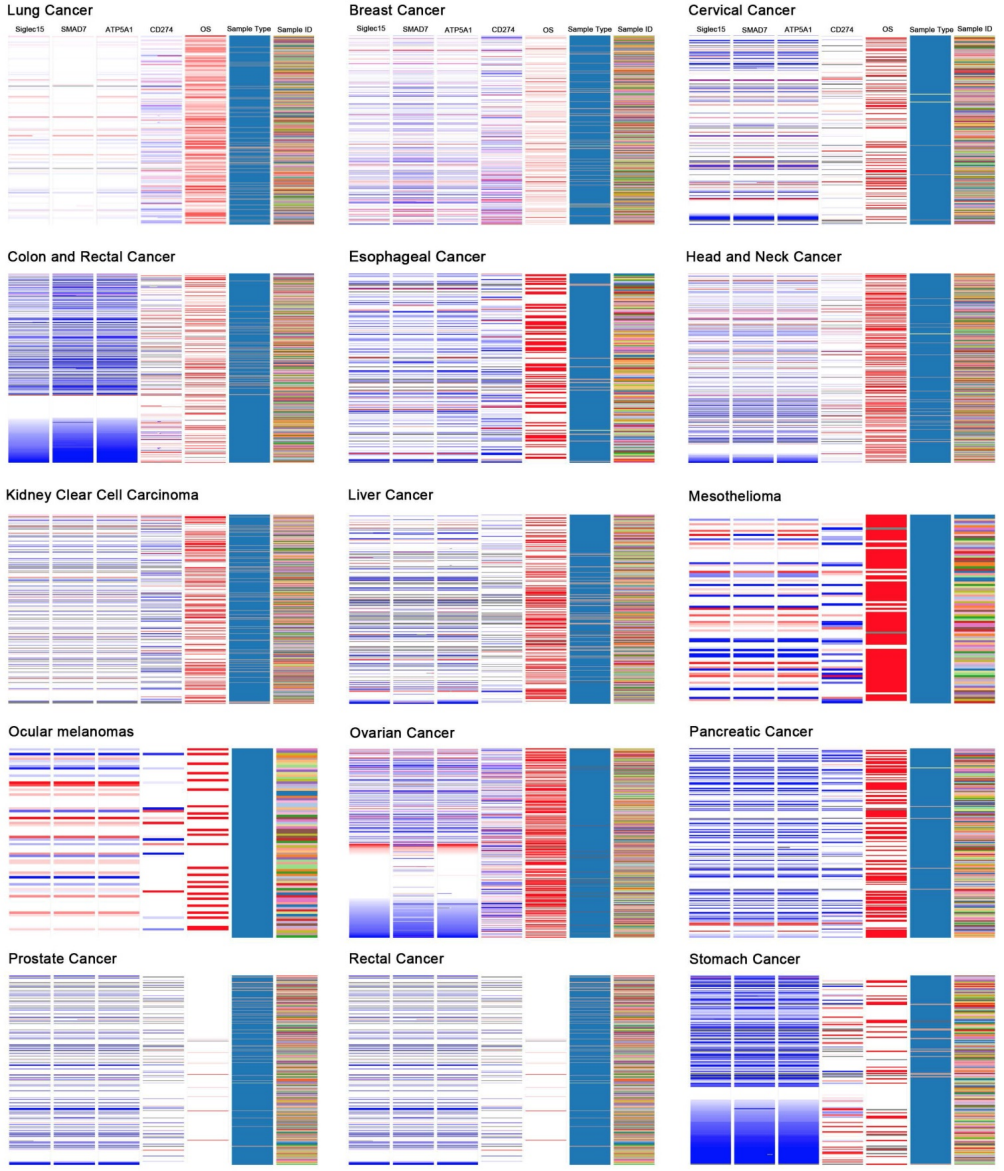
The UCSC Cancer Genomics Browser was used to explore the relationships among Siglec-15 and SMAD7, ATP5A1 and PD-L1.

Figure 6 displays the coexpression results of Siglec-15 in different cancers. According to the results, SMAD7 and ATP5A1 show similar trends, while Siglec-15 and CD274 (PD-L1) show the opposite trend. SMAD7 is a nuclear protein that binds the E3 ubiquitin ligase SMURF2 and is closely connected with the TGF-β pathway. For cutaneous melanoma patients, high Siglec-15 expression indicates a high risk of tumor aggressiveness and adverse clinical outcomes 29. The same conclusion was drawn for colon, ovarian, and endometrial cancers 30-33. ATP5A1 encodes a subunit of mitochondrial ATP synthase and plays a critical role in catalyzing ATP synthesis, participating in tumor angiogenesis and regulating tumor-related phosphorylation 34. The upregulation of ATP5A1 is widely observed in clear cell renal cell carcinoma, glioblastoma, colorectal cancer and thyroid cancer 34-37. In this study, Siglec-15, SMAD7 and ATP5A1 showed the same expression trends in tumors; thus, they may share some pathways that promote tumor aggressiveness. CD274 (PD-LI) is one of the most well-known biomarkers for tumor immunological therapy. Its inhibitor, atezolizumab, is used in bladder cancer and non-small cell lung cancer, as approved by the FDA 38, 39. In this study, we found that Siglec-15 expression is mutually exclusive from PD-L1. This result is supported by findings reported in a previous study 11. Therefore, targeting Siglec-15 may be an option for patients who are not candidates for PD-L1 immunotherapy.

In conclusion, our study suggested that Siglec-15 mRNA is upregulated in many cancers and that the differential expression of Siglec-15 leads to different prognoses. Siglec-15 mutations are widely observed in tumors and interact with different genes in different cancer types, which might explain why high Siglec-15 expression does not necessarily indicate a poor prognosis in all cancers. Siglec-15 represents a potential target for the expansion of cancer immunotherapy in some kinds of cancers.

## Supplementary Material

Supplementary tables.

## Abbreviations

TCGA: The Cancer Genome Atlas
SAGE: Serial analysis of gene expression
COSMIC: Catalog of Somatic Mutations in Cancer
Siglec: sialic acid-binding immunoglobulin-like lectin
SMAD7: SMAD family member 7
ATP5A1: ATP synthase, H+ transporting, mitochondrial F1 complex, α subunit 1, cardiac muscle
PD-L1: Programmed cell death 1 ligand 1
GEO: Gene Expression Omnibus
EGA: European Genome-phenome Archive
KIRP: Kidney renal papillary cell carcinoma
COAD: Colon adenocarcinoma
ESCA: Esophageal carcinoma
PAAD: Pancreatic adenocarcinoma
BLCA: Bladder urothelial carcinoma
PCPG: Pheochromocytoma, Paraganglioma
KICH: Kidney chromophobe
LIHC: Liver hepatocellular carcinoma
LUAD: Lung adenocarcinoma
HNSC: Head and neck squamous cell carcinoma
READ: Rectum adenocarcinoma
THCA: Thyroid carcinoma
UCEC: Uterine corpus endometrial carcinoma
CESC: Cervical squamous cell carcinoma
THYM: Thymoma
LUSC: Lung squamous cell carcinoma
PRAD: Prostate adenocarcinoma
BRCA: Breast invasive carcinoma
GBM: Glioblastoma multiforme
CHOL: Cholangiocarcinoma
STAD: Stomach adenocarcinoma
KIRC: Kidney renal clear cell carcinoma
